# Nurses’ perceptions of job security and performance: A comparative study between governmental and private hospitals

**DOI:** 10.1371/journal.pone.0318412

**Published:** 2025-03-11

**Authors:** Islam Oweidat, Ghada Abu Shosha, Raghad Al-Harazneh, Khalid AL-Mugheed, Majdi Alzoubi, Amany Anwar Saeed Alabdullah, Sally Mohammed Farghaly Abdelaliem

**Affiliations:** 1 Department of Community and Mental Health Nursing. Faculty of Nursing, Zarqa University, Zarqa, Jordan; 2 Department of Community and Mental Health Nursing Faculty of Nursing, Zarqa University, Zarqa, Jordan; 3 Faculty of Nursing, Zarqa University, Zarqa, Jordan; 4 College of Nursing, Riyadh Elm University, Riyadh, Saudi Arabia; 5 Faculty of Nursing, Al-Zaytoonah University of Jordan, Amman, Jordan; 6 Department of Maternity and Pediatrics Nursing, College of Nursing, Princess Nourah bint Abdulrahman University, Riyadh, Saudi Arabia Riyadh; 7 Department of Nursing Management and Education, College of Nursing, Princess Nourah bint Abdulrahman University, Riyadh, Riyadh, Saudi Arabia; The World Islamic Sciences and Education University, JORDAN

## Abstract

**Aim:**

Job security and performance are crucial needs for nurses, greatly influencing their motivation and commitment to work. This study seeks to examine Jordanian nurses’ perceptions of job security and employee performance and to compare these factors between government and private hospitals.

**Design:**

A descriptive, comparative cross-sectional design was used for the study.

**Methods:**

The study was conducted in two government and two private hospitals. A total of 156 nurses were conveniently sampled to participate. Data were collected using the Job Security Questionnaire and the Six-Dimension Scale of Nursing Performance.

**Results:**

The mean job security score was 3.26, indicating a moderate level of job security. The mean employee performance score was 2.68, reflecting a high level of job performance. Nurses in private hospitals had significantly higher scores in both job security (t =  -5.53, p <  0.001) and employee performance (M =  2.53, SD ±  0.53) compared to nurses in government hospitals.

**Conclusion:**

Nurses with moderate job security levels achieved high job performance scores. Private hospitals demonstrated higher job security and employee performance levels than government hospitals. Future research could investigate specific aspects of the Jordanian nursing work environment that may contribute to the differences observed in job security and employee performance.

## Background

The healthcare industry is being challenged by growing demands associated with an aging population and more access to healthcare services as the extent and complexity of health services have grown [1]. A rise in the need for nursing care has been reported in most countries around the world. In the United Kingdom, for example, the census revealed that there is a gap between the number of retiring nursing and those who are being recruited as a replacement [2]. The World Health Assembly and the Eastern Mediterranean Regional Committee are committed to strengthening the nursing profession over the years [3]. Despite this high-level commitment, the region continues to face significant issues in terms of nursing capacity. The State of World Nursing 2020 Report, which was released on World Health Day, indicates a global shortage of 5.9 million nurses, with the Eastern Mediterranean Region accounting for 17% of that shortage. While the global nurse density is 36.9 per 10,000 people, the region only has 15.6 nurses per 10,000 people [4].

Employee performance is crucial for organizational management, playing a key role in achieving a competitive advantage in the business sector [5]. In the healthcare sector, the increase in the job performance of healthcare employees, particularly nurses, reflects on the whole organization’s performance by providing high quality and efficient health services [6].

Job security is one of the basic needs of nurses and it has a great impact on their willingness to work [7]. Furthermore, job security plays an important role in improving workplace performance and the development of high-quality outputs through increasing employee retention, investment in company-specific skills and productivity, and societal effects such as social discontent, consumer confidence, and savings behavior [8,9]. Job insecurity is described by researchers as a significant cause of stress for employees, one that can significantly impact their psychological health, as well as their attitude toward their work and, ultimately, their work efficiency [10]. The term “job security” described as a consistent sense of security regarding one’s employment and salary [11]. Job security is also described as a person’s assurance that he or she will keep their current job in the future. It also relates to long-term aspirations for job progression chances [12]. If a person believes he or she has suitable permanent work and is qualified to perform it satisfactorily, he or she appears to have job security [13]. Moreover, employee job security consists of certain assurances about one’s workplace to maintain social and economic security [14].

Several studies have been looking for the most crucial criteria for employees’ performance in many professions. The majority of them emphasized the importance of job security in the workplace, with the majority of their research findings indicating a direct link between job security and employee productivity [15]. The weakening of job security has been reflected on the performance and the productivity of organizations; in addition, it possessed an impact on the degree of success regardless of the high competencies of the employees and specialization in their work field [16].

Unemployment has been a widespread concern across most nations, driven by factors such as technological advancements and an aging population [17]. As a result, firms employ a variety of strategies to downsize their workforce while remaining competitive, including restructuring, reorganization, mergers, and acquisitions. Taken altogether, these frequent changes in organizational structures for many organizations heightened the workers’ sense of job insecurity [18].

On the other hand, the healthcare industry is being challenged with unprecedented challenges and competitive pressures. The rising costs of healthcare, advancing technology, an aging populace with a wide range of needs and care requirements, and new sorts of illnesses and diseases have all increased the demands on hospitals and their staff [19]. Furthermore, hospitals would value employees who have a positive attitude toward their professions and who support the hospital’s attempts to improve the quality of healthcare services. This could improve patients’ perceptions of their healthcare experience, boosting the effectiveness of a hospital’s marketing efforts and, ultimately, its performance as a service company [20].

Nurses are the heart and backbone of all hospital facilities and the cornerstone of healthcare in Jordan. In addition, nurses can contribute to putting the country in a competitive position and making a name for itself in the healthcare industry. The feeling of job security amongst nurses is mostly associated with their prospects and their predicted successes in the future. When nurses feel insecure about their jobs, it affects the efficiency of their work, i.e., the provision of high-quality safe patient care in the hospital [21]. Hence, the study can be particularly important for two groups of stakeholders: first; healthcare organizations, which, after gaining knowledge about the negative effects of low job security and the costs associated with them, can take appropriate steps to ensure job security. This could occur by developing policies and procedures, thereby improving employees’ “job performance”. Second; the employees, who, after experiencing job security, exhibit less burnout and stress, become more satisfied with their jobs and have better job performance. This will be reflected by avoiding a variety of health problems and getting better quality care. In addition, this could improve patients’ perceptions of their healthcare experience, boosting the effectiveness of a hospital’s marketing efforts and, ultimately, its performance as a service company [20].

### Job security

Job security is defined as the possibility that the person will save his or her job, with little chance of becoming unemployed [22]. Another definition of job security is “the feeling of having a good job and being confident in the employment’s continuation regardless of the presence or absence of external threat factors such as the external environment’s recession” [23].

A study found that over half of the staff nurses had a moderate perception of job security, whereas less than half reported moderate levels of work alienation. The results indicated a statistically significant negative correlation between nurses’ perceptions of job security and their experiences of work alienation [24]. In a separate study, Sulaiman et al., reported that nurses generally had high levels of job security, while levels of Leader Empowering Behaviors (LEB) and Core Self-Evaluations (CSE) were moderate [25]. This study also revealed significant positive relationships between CSE, LEB, and job security. Dhanpat et al., found a link between retention factors and job security, identifying retention factors as key predictors of job security [26]. Among these, training and development emerged as the strongest predictors of job security for nurses, offering valuable insights into strategies for retaining nursing professionals.

Job insecurity is a common and costly issue in healthcare, affecting nurses and other health professionals [21]. Nurses’ sense of job security often depends on their expectations for future success and performance [27]. When nurses feel insecure in their roles, it negatively impacts both hospital efficiency and patient care [28]. The most notable effect of job insecurity on nurses is reduced job satisfaction, which contributes to higher turnover rates [13]. This issue is particularly concerning given the current nursing shortage, which affects the effectiveness of healthcare delivery [29]. Additionally, job insecurity among nurses has been associated with health deterioration, increased burnout, stress, vulnerability, and lower job satisfaction [30]. Furthermore, job insecurity has led many nurses to seek opportunities abroad for better pay and quality of life [23].

### Job performance

Employee performance is defined by how effectively a staff member fulfills job responsibilities, completes assigned tasks, and conducts themselves in the workplace 30. Performance is evaluated based on the quality, quantity, and efficiency of the work produced [31]. Employee performance is also defined as a member of an organization contributing to achieving the goals of the organization [32,33].

In recent study, the results showed that nursing performance was significantly influenced by recruitment and selection, training and development, rewards and benefits, performance appraisal, strategic planning, and organizational culture [34]. Mamdouh et al. found that nurses demonstrated unsatisfactory levels of knowledge and performance, with a statistically significant correlation between the two [35]. In another study, Kahya and Oral reported that, while clinical and professional skills were considered the most important categories, “working systematically” in the contextual category received the highest scores [36].

Nurses form the backbone of patient care, and their sense of job security and performance has direct implications for healthcare quality, patient safety, and organizational efficiency. In our country, the healthcare system is undergoing significant changes, including workforce restructuring, increasing demand for healthcare services, and global competition for skilled professionals. These changes may impact nurses’ job security and, consequently, their performance. The main purpose of this study is to identify Jordanian nurses’ perceptions of job security and employee performance, and to compare these variables between governmental and private hospitals.

### Research questions

1. What level of job security do nurses believe to prevail in Jordanian hospitals?
2. What level of employee performance do nurses perceive themselves to be achieving in Jordanian hospitals?
3. Which hospitals in Jordan have the highest employee performance and job security for nurses? Which hospitals are better, either governmental or private?

## Methodology

### Study design

This study is a descriptive cross-sectional study including descriptive correlation and descriptive comparative strategies

### Settings

The study was conducted in two governmental hospitals and compared with two private hospitals. Governmental hospitals are (1) Al Bashir Hospital (2) Prince Hamza Hospital. Al Bashir Hospitals is composed of four distinct hospitals including Medical Hospital, Surgical Hospital, Maternity and Pediatric Hospital, and Emergency Hospital. Al Bashir Hospitals have a total bed capacity of 1,088 beds and a total number of 1200 nurses, making it the largest governmental and referral hospital in Jordan. Second, Prince Hamza Hospital has a total bed capacity of 436 beds and 450 nurses. These hospitals are chosen because of the large number of patients catered to which reflects the healthcare demand that must be met by competent, safe and quality nursing management. [37]

On the other hand, the private hospitals are the Islamic Hospital it contains a comprehensive emergency and accident medicine center, more than 10 operating rooms supplied with cutting-edge equipment, daily special rooms, and several royal rooms and suites. It has a total of 423 beds (273 fixed beds and 150 mobile beds) and over 450 qualified nurses. Istishari Hospital has a capacity of 108 beds. It is committed to provide the highest quality patient care. Thus, these two hospitals were chosen from the private sector because of the large number of patients catered to which reflects the healthcare demand and the high-level matching between the above governmental hospitals and private hospitals.

### Sampling technique

The sample size calculation was based on a correlation test statistical method. Using the G * Power software (version 3.1.9.4), the sample size was determined to achieve a power level of 0.95, with an alpha level (Type I error) set at 0.05 and a medium effect size of 0.3. The correlation test assesses the strength and direction of a relationship between two variables, and the specified parameters allow for a high probability (95%) of detecting a statistically significant correlation if one truly exists in the population. The minimum sample size required was 134. This number was increased to 160 to overcome the possibility of missing data or possible nonresponse rates.

The participants were selected from the chosen governmental and private hospitals using a convenience sampling technique. Inclusion criteria for the participating nurses were: (1) Jordanian nurses; (2) nurses interested to participate in the research; (3) nurses who have a work experience of at least 1 year due to the fact that the employee will be exposed to trial before employment during their first year of employment—and that certain employment contracts in government hospitals are only for one year—they won’t feel secure in their position, and fulltime employment as a hospital nurse; and (4) having bachelor’s degree or above in educational level. While the exclusion criteria were: (1 nurses who are unwilling to participate in the study; and (2) Nurses who are in a trial phase, have recently been hired, or have an employment contract that lasts just one year and won’t be renewed.

### Instruments

Tool I: A self-administered questionnaire was used to find out to which degree the nurses are secured on their job. This tool included two parts:

Demographic data sheet includes assessment of the personal and occupational characteristics of the nurses in terms of gender, age, educational status, marital status, working area, monthly income, years of experience in nursing, years of experience of current hospital, years of experience in the current department.

Part two comprises Job security questionnaire which is a 36-item questionnaire that was developed by Sokhanvar et al.,^13^. The responses for this scale were rated on a 5-point Likert scale from 1 “Strongly disagree”) to 5 (“Strongly agree”). The questionnaire’s mean score for job security was 3.10 ±  0.38, as suggested by the authors, were categorized and interpreted as follows: scores 0–2: very low job security; scores 2.1–2.75: low job security; scores 2.76–3.50: moderate job security; scores 3.51–4.25: high job security; and scores 4.26–5: very high job security. The overall Cronbach alpha of Job security was 0.78

Tool II: The Six-Dimension Scale of Nursing Performance (Six-D Scale) which includes 52 items questionnaire that was developed by Schwirian, was used to assess nurses’ performance [38]. The respondents were asked to answer the research questions by ticking their level of agreement or disagreement regarding the activities in which nurses engage with varying degrees of frequency and quality and they were divided into 2 columns. column A consists of 42 items and it is designated to assess the frequency of job performance. The responses are (1) Not expected in this job, (2) Never or seldom, (3) Occasionally, and (4) Frequently. Column B consists of 52 items and is used to assess the quality of job performance. The responses are (1) Not very well, (2) Satisfactory, (3) Well, and (4) Very Well. The total scores were calculated according to the average of the items per sub-scale. The Likert scale is divided as follows; low score if the level is under 2, average scores are (2-2.5), and above average scores are (2.5-4) [39, 40]. Since the definition of job performance in this study focused on how well a staff member performed their duties, we were unable to present all the data regarding frequency and quality since the table and data were too large. As a result, this study will present results related to quality; results pertaining to frequency will be presented in a subsequent study. The Six Dimension Scale of Nursing Performance (Six-D Scale) was reliable (0.844 - 0.978).

### Data collection procedure

After the researcher had obtained the ethical approvals, the researcher visited the departments of data collection (ER, medical-surgical male and female, labor, postpartum, adult and pediatric ICU), and introduced herself to the nurse managers, outlining the objectives of the study and how the data would be collected.

Data collection started on (01 Aug 2022) and completed on (20 Sep 2022). The final sample size included 156 participants. A list of potential participants was requested from nurse managers based on the inclusion-exclusion criteria. The researcher contacted nurses who met the study’s eligibility requirements and extended an invitation for them to participate, the purpose, potential hazards, and participant responsibilities were all discussed by the researcher. To enhance communication between the researcher and participants, a WhatsApp group was created. Online forms of study questionnaires were done by the researcher utilizing Google forms. All forms were in English language. A link that includes the consent form, cover letter, and study objectives was sent to participants through the WhatsApp group.

Nurses who agreed to fill out the online version were given the option to save and submit it later, but they were reminded to complete the questionnaires before the expiry date of the link which will be after seven days. The researcher visited the chosen hospitals several times to talk with staff members working various shifts. The data collection was ended once the target sample size has been reached by the response rate.

### Data analysis

Descriptive statistics including percentages, frequencies, means and standard deviations were used to describe the socio-demographic variables and findings on job security and employee performance. An independent sample t-test was performed to compare the means of job security levels and nurses’ performance among private and governmental hospitals. The assumptions of each test were checked and no violation was detected. A significance level of p <  0.05 was set for all analyses.

### Ethics approval

The study was conducted according to the guidelines of the Declaration of Helsinki and approved by the Institutional Review at all sites. The ethical approval from Zaraq University of Jordan was obtained with the number of 6/2022. Besides, the ethical approval statements from the selected universities were granted. Written informed consent was obtained from all participants.

### Results

The findings demonstrated a divergence in gender. The females were 66% (103 respondents) of the sample, and the males were 34% (53 respondents). Regarding marital status, most respondents were married (n = 97; 62.2%), and (n = 51, 32.77%) were single nurses. Regarding educational background, 82.1% of the respondents had a bachelor’s degree (128 respondents), while 16% had a master’s degree (25 respondents). The lowest percentage of respondents was doctoral degree holders, who comprise 1.9% of the total three nurses. There were 38 nurses from Al -Bashir Hospital, 38 nurses from Prince Hamza Hospital, 50 nurses from Islamic Hospital, and 30 nurses from the Istishari Hospital. Table 1

**Table 1:** Demographic characteristics of the sample (n = 156).

Variables	Category	n	(%)
Gender	Male	53	34%
	Female	103	66%
Marital Status	Single	51	32.7
	Married	97	62.2
	Divorced	6	3.8
	Widowed	2	1.3
Educational Attainment	Bachelor	128	82.1
	Master	25	16
	PhD	3	1.9
Area of work	Al -Bashir Hospital	38	24.4
	Prince Hamza Hospital	38	24.4
	Islamic hospital	50	32.1
	Istishari hospital	30	19.2

The results in Table 2 show that the average respondent age was 33 years, with 7.6 years’ variation between the nurses. On average, the nurses were well experienced in the field as they have ten years of experience and they stayed 7.3 years in the same hospital. The mean value of their experience in the same department (unit) was 6.23 years. Finally, the average of their monthly salary was 577 Jordanian dinars equivalent to about $813.87 USD, with a gap of 181 Jordanian more or less than the average.

**Table 2:** Demographic variables of continuous data.

	Mean	SD ± (Standard Deviation)
Age	33	±7.6
Years of Experience Working as a Registered Nurse	10.1	±7.8
Years of Experience Assigned in Place of work	7.34	±5.6
Years of Experience Assigned in This Unit	6.23	±5.4
Monthly Income (Jordanian Dinar)	577.45	±181.5

The mean value of the job security score was 3.26 as a central tendency measurement of the variable, which reflects a moderate level of job security. the median value, which came in the middle of all responses, was 3.31, and the standard deviation was low compared to the mean value (±0.43). the “overall job security score percentage” (mean score) value were 65.36, which was high. the employee performance score average was 2.68, reflecting a high level of job performance. last, the average employee performance score percentage (mean score) was 67.10, which was high. Table 3

**Table 3:** Job Security and Employee Performance levels (n = 156).

	Mean	Median	Standard deviation	Min	Max
Job Security Score	3.26	3.31	0.43	1.77	4.63
Overall Job Security Score Percentage (Mean Score)	65.36	66.30	8.78	35.40	92.60
Employee Performance Score	2.68	2.73	0.54	1.00	3.99
Employee Performance Score Percentage (Mean Score)	67.10	68.37	13.58	25.00	99.75

The means of two unrelated groups on the same continuous dependent variable were compared using the independent-samples t-test. The main value of the independent t-test aimed to compare the governmental and private hospitals in Jordan regarding job security among nurses (Table 4). Nurses working in private hospitals (3.44 ± 0.37) had significantly higher Overall Job Security than nurses working in governmental hospitals (3.08 ± 0.43) (t =  -5.53, p <  0.001). Similar results of nurses working in private hospitals (2.82 ± 0.51) having significantly higher levels of employee performance than nurses working in governmental hospitals (2.53 ± 0.53) were found (t =  -3.50, p <  0.001).

**Table 4:** Job Security and Employee Performance of Nurses Working in Governmental and Private Hospitals.

Work Place		N	Mean ± Std. Deviation	P Value
Score				
Overall Job Security Score	Governmental	76	3.08 ± 0.43	<0.001
	Private	80	3.44 ± 0.37	
Overall Employee Performance Score	Governmental	76	2.53 ± 0.53	<0.001
	Private	80	2.82 ± 0.51	
Percentage Mean Score				
Overall Job Security Score Percentage Mean Score	Governmental	76	61.70 ± 8.61	<0.001
	Private	80	68.84 ± 7.47	
Overall Employee Performance Score Percentage Mean Score	Governmental	76	63.33 ± 13.44	<0.001
	Private	80	70.68 ± 12.80	

Years of experience as a registered nurse (r =  -0.18, p <  0.05), years of experience in the current hospital (r =  -0.20, p <  0.05), and years of experience in the current unit (r =  -0.19, p <  0.05) had a weak negative but significant relationship with employee performance. Table 5

**Table 5:** Job Security and Employee Performance of Nurses Based on Age and Seniority.

	Overall Job Security Score		Overall Employee Performance Score	
r	P. value	r	P. value
Age	0.083	0.303	-0.156	0.051
Years of Experience working as a Registered Nurse	0.074	0.359	-0.175	0.029
Years of Experience Assigned in the Place of Work	0.038	0.636	-0.200	0.012
Years of Experience Assigned in this unit	0.031	0.698	-0.191	0.017
Monthly income	0.093	0.251	-0.131	0.103

## Discussion

The study revealed a moderate level of job security and a high level of employee performance among nurses. This indicates that the nurse participants effectively carried out their nursing skills, responsibilities, and duties. Similar findings were reported in other studies on Jordanian nurses, which also observed high levels of job security and performance [26,39]. These findings underscore the effectiveness of Jordan’s current healthcare system structure in maintaining job security for nurses, despite persistent challenges like staff shortages, high turnover rates, and migration issues.

Suleiman et al., further emphasized that job security perceptions were higher among nurses with permanent contracts compared to those hired under performance- or productivity-based models [25]. Additionally, the high employee performance levels suggest that nurses in Jordanian hospitals can uphold patient safety and care quality, even with increasing challenges such as complex patient conditions, reliance on advanced technologies, and heightened stress and anxiety levels [1].

The results showed that nurses working in private hospitals had significantly higher levels of job security and employee performance compared to nurses working in governmental hospitals. As to the author’s knowledge, this study is the first to generate empirical data comparing job security and employee performance in different hospitals settings in Jordan. The results suggested the existence of structural and organizational differences between government and private hospitals that brought about variations in scores on job security and employee performance. Private hospitals often provide better funding, allowing for modern facilities, more resources, and favorable employment terms, such as permanent contracts and competitive salaries, which enhance job security [25]. For instance, Albasal and colleagues in a cross-sectional study found that government and private hospitals differed in the levels of structural and psychological empowerment, which in turn led to differences in organizational commitment, job satisfaction, nurse performance, and in extension, job security [41]. Also, training opportunities, quality supervisor support, and recognition programs are more prevalent in private hospitals, fostering professional development and workplace satisfaction [42]. In contrast, governmental hospitals face challenges such as limited budgets, higher workloads, and bureaucratic inefficiencies, which can negatively affect staff morale and stability. Addressing these disparities through targeted policies could improve job satisfaction and performance across hospital types, ensuring a sustainable nursing workforce in Jordan [43].

### Implications for Nursing Practice and Administration

The employees in the organization expected to continue their job with certainty without unexpected sudden job loss. The job safety in the workplace makes employees enthusiastic in doing their job duties results more employee performance. Employee job security is regarded as vital element in the organization in increasing their job performance which ultimately fosters organization’s total productivity. Moreover, employee job security has significant role to improve workplace performance as well as production of quality outputs. Few decades ago, several studies have been conducted and found positive result on employee job security and performance relationship [13,30].

Employees do not hesitate to work hard in fulfilling organization’s targets when they are entrusted that their job is secured. Certainty of employees’ job continuation makes them committed to the organization and in turn they perform more job duties. As such, it is imperative for nurse managers and hospital administrators to ensure that interventions are in place to make nurses feel secure about their job and offer support to improve their performance and productivity. A collective benefit of a healthy nursing workforce is the primary organizational advantage of ensuring that individual nurses are satisfied with their jobs and performing up to standards [44, 45].

### Implications to Research

Nurse researchers can significantly contribute in the conceptualization, development and implementation of future research in investigating the organizational factors influencing job security and employee performance of Jordanian nurses. Specifically, future research can explore why levels of job security and employee performance are different between nurses working in governmental and private hospitals, whether or not such differences impact patient-, nurse- and organizational-outcomes, and to what extent such differences impact the overall safety and quality of patient and nursing care.

### Strengths and Limitations

The strengths of the study are (1) adequate sample size, (2) adequate power, (3) and exploration of differences between the job security and employee performance of nurses working in Jordanian government and private hospitals. However, the limitations of the study were (1) the cross-sectional research design, and (2) the involvement only of nurses based in hospitals. First, job security and employee performance are not static variables; that is, while levels might have been high at the time of data collection, there is no assurance that levels are always high.

A longitudinal study design will be more appropriate to detect changes on the levels of job security and employee performance over time. Second, nurses working in other healthcare settings such as clinics, primary health centres, and the community might have totally different levels of job security and employee performance. As such, generalizability of results is limited to nurses working only in hospital settings and not to other cohorts.

## Conclusions

Nurses working in Jordanian hospitals demonstrated a moderate level of feeling about having job security and a high level of employee performance. Nurses with high levels of job security obtained high scores on job performance. Female and senior nurses had significantly lower employee performance than their counterparts. Nurses working in private hospitals had significantly higher employee performance than nurses working in government hospital. Nurse managers and administrators must establish policies and employment contracts that clearly assure staff—both newly hired and existing—that their jobs are secure, protected from arbitrary termination, and subject to due process. Managerial, peer, and organizational support systems should be strengthened to improve nurse productivity and performance, as this significantly impacts organizational outcomes and ensures patient safety and care quality. Efforts should also focus on addressing disparities between government and private hospitals by improving structures and organizational cultures to ensure that nurses feel secure and perform well regardless of their workplace. Creating equitable environments across hospital types will help reduce observed differences and promote a sustainable nursing workforce. Future research should explore specific characteristics of work environments that contribute to these disparities to inform targeted interventions.

## Supporting information

S1 Data Study_dataset. (SAV)
